# Recurrence of anxiety disorders and its predictors in the general population

**DOI:** 10.1017/S0033291721002877

**Published:** 2023-03

**Authors:** Willemijn Scholten, Margreet ten Have, Carmen van Geel, Anton van Balkom, Ron de Graaf, Neeltje Batelaan

**Affiliations:** 1 Amsterdam UMC, Vrije Universiteit, Psychiatry, Amsterdam Public Health Research Institute, Amsterdam, The Netherlands; 2 GGZ inGeest Specialized Mental Health Care, Amsterdam, The Netherlands; 3 Netherlands Institute of Mental Health and Addiction, Utrecht, The Netherlands

**Keywords:** Anxiety disorders, course, general population, predictors, recurrence

## Abstract

**Background:**

Anxiety disorders frequently recur in clinical populations, but the risk of recurrence of anxiety disorders is largely unknown in the general population. In this study, recurrence of anxiety and its predictors were studied in a large cohort of the adult general population.

**Methods:**

Baseline, 3-year and 6-year follow-up data were derived from the Netherlands Mental Health Survey and Incidence Study-2 (NEMESIS-2). Respondents (*N* = 468) who had been in remission for at least a year prior to baseline were included. Recurrence was assessed at 3 and 6 years after baseline, using the Composite International Diagnostic Interview version 3.0. Cumulative recurrence rates were estimated using the number of years since remission of the last anxiety disorder. Furthermore, Cox regression analyses were conducted to investigate predictors of recurrence, using a broad range of putative predictors.

**Results:**

The estimated cumulative recurrence rate was 2.1% at 1 year, 6.6% at 5 years, 10.6% at 10 years, and 16.2% at 20 years. Univariate regression analyses predicted a shorter time to recurrence for several variables, of which younger age at interview, parental psychopathology, neuroticism and a current depressive disorder remained significant in the, age and gender-adjusted, multivariable regression analysis.

**Conclusions:**

Recurrence of anxiety disorders in the general population is common and the risk of recurrence extends over a lengthy period of time. In clinical practice, alertness to recurrence, monitoring of symptoms, and quick access to health care in case of recurrence are needed.

## Introduction

Current knowledge on recurrence rates predominantly comes from studies in clinical populations (e.g. primary care and mental health care). These studies show substantial recurrence rates across various types of anxiety disorders. For example, in the Harvard/Brown Anxiety Disorders Research Program (HARP), 711 patients with anxiety disorders from clinical treatment facilities were prospectively followed for 12-years (Bruce et al., 2005). Subsequently, recurrence rates were calculated in patients who had achieved remission since intake, and this yielded recurrence rates varying from 39% for patients with social phobia to 58% for panic disorder with agoraphobia (PDA). In another longitudinal study, the recurrence rates of anxiety disorders were studied in a largely clinical sample (*N* = 429) from the Netherlands Study of Depression and Anxiety (NESDA), consisting of people with a history of anxiety disorders, but with no current anxiety disorder at baseline (Scholten et al., 2013). A recurrence rate of 23.5% within 2 years after baseline assessment was found, with no significant difference between panic disorder with or without agoraphobia, social anxiety disorder and generalized anxiety disorder, which was almost similar to recurrence in another sample from the same cohort (Scholten et al., 2016). Even after responding to psychological treatment, relapse rates are still high. In a recent meta-analyses of on relapse after cognitive behavioral therapy (CBT) in anxiety disorders (nine studies; *N* = 532 patients), on average 23.8% of patients experienced relapse following completion of CBT (Lorimer, Kellett, Nye, & Delgadillo, 2021).

Predictors of recurrence are also mostly investigated clinical samples. Clinical characteristics, such as comorbid depressive and different anxiety disorders, and higher levels of dysfunctioning, were found to be predictive of recurrence of the previous anxiety disorder (Bruce et al., 2005; Fava et al., 2001a, 2001b; Heldt et al., 2010; Mavissakalian & Guo, 2004; Pagano et al., 2007; Ramsawh, Weisberg, Dyck, Stout, & Keller, 2011; Rodriguez, Bruce, Pagano, & Keller, 2005; Taylor, Jakubovski, & Bloch, 2016; Weisberg, Machan, Dyck, & Keller, 2002). Furthermore, vulnerability characteristics such as neuroticism, higher anxiety sensitivity and parental substance abuse were identified as predictors (Calkins et al., 2009; Donovan, Glue, Kolluri, & Emir, 2010; Pagano et al., 2007; Scholten et al., 2013; Taylor et al., 2016). Only two of these studies analyzed a broad range of sociodemographic, clinical and vulnerability characteristics in multivariable analyses (Scholten et al., 2013; Taylor et al., 2016). Both studies identified ‘anxiety sensitivity’ (the tendency to respond to anxiety symptoms fearfully) and higher levels of dysfunctioning as predictors of recurrence in multivariable analyses. In the study by Taylor et al. (2016), being single, smoking and treatment with benzodiazepines were additionally associated with recurrence. Univariate analyses are needed to find predictors which can identify people at risk of recurrence. To be able to find targets for interventions to prevent recurrence, multivariable analysis is needed, because it identifies the predictors which independently predict recurrence. In conclusion, the risk of recurrence in clinical populations is substantial for all anxiety disorders, with several identified predictors of recurrence.

Findings from clinical samples may not be generalizable to the general population, since (i) not every person with an anxiety disorder in the general population seeks or receives treatment and (ii) people who seek help in clinical care often have more severe disorders (Ten Have, Nuyen, Beekman, & De Graaf, 2013). Furthermore, only a few studies provide data on long-term recurrence rates, and thus far it remains unclear whether this risk remains over time. To gain knowledge on the naturalistic course of anxiety disorders, and to enable identification of people at high risk of recurrence, long-term data on recurrence and on predictors of recurrence in the general population are needed. It is known that anxiety disorders are common in the general population (De Graaf, Ten Have, Van Gool, & Van Dorsselaer, 2012; Hardeveld et al., 2013; De Jonge et al., 2016; Kessler et al., 2006; Ormel et al., 2008; Ruscio et al., 2017; Stein et al., 2017), but only two studies in the general population studied recurrence and its predictors in anxiety disorders. However, these were in specific samples, and therefore generalizability may be limited. In the first study by Calkins et al. (2009), predictors of both onset and recurrence were studied together in a population-based sample of women between 36 and 45 years old with and without previous anxiety disorders. In this study, a total of 643 women participated, of whom 4.5% reported a new onset or recurrence of anxiety (all anxiety disorders except for specific phobias) during the 3-year follow-up period. Using a multivariable model, only history of an anxiety disorder and anxiety sensitivity were significant predictors in this community sample of women. A disadvantage of this study is that it did not differentiate between predictors of first-onset and recurrence. The second general population study specifically focused on recurrence and its predictors, but was limited to panic disorder and PDA (Nay, Brown, & Roberson-Nay, 2013). In this study, 3-year follow-up data from the National Epidemiologic Survey on Alcohol and Related Conditions were used. Recurrence of panic disorder within 3 years was 12% *v.* 21.4% for PDA. Baseline diagnosis of generalized anxiety disorder, agoraphobia, nicotine dependence, female sex, younger age, and younger age of onset and having a major financial crisis significantly predicted recurrence.

To summarize, assessing recurrence of anxiety disorders long-term and studying their predictors is a relatively unexplored research area in general population studies. Previous studies mainly included clinical samples rather than population samples. The few existing studies in the general population included specific samples or only investigated a few specific anxiety disorders, had substantially shorter follow-up data (3-years), and did not investigate lifelong risk of recurrence. The present study addressed these gaps in the literature by investigating recurrence and predictors of recurrence of anxiety disorders by using data from three waves of the Netherlands Mental Health Survey and Incidence Study-2 (NEMESIS-2), a nationally representative adult population survey (De Graaf, Ten Have, & van Dorsselaer, 2010), and conducting univariate and multivariable analyses. Our research questions are (1) What is the cumulative recurrence rate of anxiety disorders (social anxiety disorder, panic disorder with or without agoraphobia, agoraphobia alone and generalized anxiety disorder) in the general population? (2) What are predictors for time to recurrence, using a broad range of general vulnerability, physical health and mental health predictors?

## Methods

### Design

Baseline, 3-year and 6-year follow-up data were derived from NEMESIS-2, a prospective psychiatric epidemiological population study, conducted between November 2007 and June 2015. In brief, 6646 persons of the Dutch population aged 18–64 were interviewed face-to-face, with laptop computer assistance. The response rate was 65.1% and the average duration of the interview was 95 min. The sample was based on a multistage, stratified random sampling of households with one respondent randomly selected in each household. The sample was nationally representative, although only younger subjects were somewhat underrepresented (De Graaf et al., 2010).

All respondents from the first wave (*T*_0_) were approached for follow-up (*T*_1_), 3 years later, from November 2010 to June 2012, and 5303 persons could be interviewed again (response rate 80.4%, with those deceased excluded; duration 84 min). All *T*_1_ respondents were approached for second follow-up (*T*_2_), 3 years after *T*_1_, from November 2013 to June 2015. This time, 4618 persons were interviewed again (response rate 87.8%; duration: 83 min). Attrition between *T*_0_ and *T*_2_ was not significantly associated with all 12-month mental disorders at *T*_0_, after controlling for sociodemographics, except for bipolar disorder (de Graaf, van Dorsselaer, Tuithof, & Ten Have, 2015).

The Medical Ethics Review Committee for Institutions on Mental Health Care (METIGG) approved the study. Respondents were informed about the study aims and provided written informed consent at each wave. A more detailed description of the design and the respondents of NEMESIS-2 can be found elsewhere (De Graaf et al., 2010).

### Sample

The sample for this study consisted of respondents remitted from any anxiety disorder (i.e. they had a lifetime anxiety disorder but not a 12-month anxiety disorder) at baseline who participated in at least one follow-up wave, following Ten Have et al. (2018). The number of participants was 468 at baseline (*T*0), 468 at 3-year follow-up (*T*1), and 411 at 6-year follow-up (*T*2). Past or current depressive disorders or alcohol use disorders were allowed. Of these 500 respondents, 10 respondents were excluded due to missing values of the variable ‘recency of the anxiety disorder’ (number of years since remission of the last anxiety disorder), and 22 respondents were excluded because of a lifetime bipolar disorder or schizophrenia at baseline. In line with comparable NEMESIS-studies on anxiety disorders (among others Bosman et al., 2019; Ten Have et al., 2021), persons with schizophrenia and bipolar disorder were excluded, because these disorders in itself can have a fluctuating course in time and cause severe symptoms that can be difficult to distinguish from anxiety disorders. This resulted in a final sample of 468 respondents. Analyses restricted to the remitted anxiety disorder group (*n* = 468) were not weighted as we were seeking to explain, rather than describe, the interrelationships between risk indicators and time to recurrence of anxiety disorders in a specific subsample of the total population.

### Measures

#### Remitted anxiety disorder at baseline

Remitted anxiety disorder, i.e. the presence of at least one lifetime anxiety disorder >12-months prior to baseline, was assessed with the Composite International Diagnostic Interview (CIDI) version 3.0 (Kessler & Bedirhan Üstün, 2004). The CIDI 3.0 measures DSM-IV disorders, including the age of onset of the disorder and the age of recency of the disorder, and is a fully structured lay-administered diagnostic interview that was originally developed in the World Mental Health Survey Initiative. The CIDI 3.0 has a generally good validity for common mental disorders (Kessler & Bedirhan Üstün, 2004).

#### Recurrence

The primary outcome variable was the presence of any anxiety disorder during the 6-year follow-up period, further called recurrence. To determine time to recurrence, we added up the number of years since remission of the last anxiety disorder until baseline (recency of the anxiety disorder) plus the number of years from baseline to recurrence in the 6-year follow-up. In case that the recurrence occurred during the period between two interviews (on average 3 years), we assumed that the recurrence took place halfway (1.5 years).

The reason for including not only the recurrence of the index disorder, but also the occurrence of other anxiety disorders, is that transitions to other anxiety disorders frequently occur (Scholten et al., 2013). Recurrence was assessed by data of the CIDI interviews at *T*1 and *T*2 concerning the presence of anxiety disorders (yes/no) at any time in the period since the last interview.

#### Predictors and baseline characteristics

To study predictors of time to recurrence of anxiety disorders, a broad set of variables was used. All predictors were assessed at *T*0, except for parental psychopathology (assessed at *T*1). Predictors and other baseline characteristics are presented in Table 1.

**Table 1.** Baseline characteristics of respondents with remitted anxiety disorder (*n* = 468), in percentages or means with standard errors (s.e.)

	*n*	% or mean (s.e.)
Sociodemographic characteristics		
Female gender	302	64.5
Age at interview	468	46.0 (0.54)
Education		
Lower secondary	141	30.1
Higher secondary	156	33.3
Higher professional, university	171	36.6
Living without partner	159	34.0
No paid job	130	27.8
Not enough income to live on	44	9.6
Vulnerability characteristics		
Any childhood abuse	215	46.7
Any negative life event (<12 months)	237	51.5
Parental psychopathology^a^	212	45.3
Neuroticism	460	3.6 (0.13)
Physical health and functioning		
Any chronic physical disorder	204	44.3
Number of chronic physical disorders	460	0.5 (0.04)
BMI	467	25.2 (0.20)
Physical dysfunctioning scale^b^	468	19.9 (0.89)
Mental dysfunctioning scale^b^	468	18.3 (0.68)
Clinical characteristics of anxiety disorder		
Age of onset anxiety disorder	465	19.9 (0.58)
Two or more anxiety disorders	70	15.0
Recency: number of years since remission	468	11.0 (0.47)
Comorbid mental disorders		
Any mood disorder		
Never	244	52.1
Remitted	184	39.3
Current	40	8.6
Any alcohol use disorder		
Never	382	81.6
Remitted	66	14.1
Current	20	4.3
Health care management		
General medical care	56	12.2
Mental health care	42	9.1
Psychotropic medication	64	13.9

Ref, reference category.

a Assessed at the second wave, *T*_1_.

b This scale ranges from 0 (high functioning/good health) up until 100 (low functioning/ill health).

*Sociodemographic variables:* Gender, age, education (higher professional, university; higher secondary; and lower secondary), living situation (partner yes/no), job status (paid job yes/no), and household income (i.e. enough income to live on yes/no) were included as sociodemographic variables.

*Vulnerability characteristics:* The presence of childhood abuse was determined by assessing whether, before the age of 16, one had experienced emotional neglect, psychological abuse or physical abuse on ⩾2 occasions, or sexual abuse on ⩾1 occasion. The NEMESIS Childhood abuse questionnaire has been used in the NESDA (e.g. Hovens, Giltay, Spinhoven, van Hemert, and Penninx, 2015). This questionnaire shows a high similarity with the Childhood Trauma Interview (Fink, Bernstein, Handelsman, Foote, & Lovejoy, 1995), which is a reliable and valid method for brief assessment of multiple dimensions of childhood interpersonal trauma (Hovens et al., 2012). The presence of any out of 10 negative life events in the previous 12 months (yes/no), such as death of a relative or friend, divorce, and financial difficulties, based on Brugha, Bebbington, Tennant, and Hurry (1985). Parental psychopathology was measured by self-report by the respondent with a self-constructed questionnaire which was also used in NEMESIS-1 (De Graaf et al., 2010). Parental psychopathology refers to one or two biological parent(s) ever having been treated by a psychiatrist, or hospitalized in a mental institution, or ever having exhibited one or more of the following problems: severe depression, delusions or hallucinations, severe anxiety or phobias, alcohol abuse, drug abuse, regular problems with the police and suicidal behavior. Neuroticism was measured with the 12-item version of the ‘Eysenck Personality Questionnaire’, the EPQ-Revised Short Scale (Eysenck, White, & Eysenck, 1976, 1985).

*Physical health and functioning:* To assess physical health, the presence of chronic physical disorders in the previous 12 months (0–17 chronic physical disorders treated or monitored by a medical doctor), was identified with a standard checklist. In addition, body mass index (BMI) was generated by self-reported height and weight. Functioning was assessed over the past 4 weeks and was based on the Medical Outcomes Study Short Form Health Survey (Ware & Sherbourne, 1992), consisting of a higher level of physical functioning scale and a mental functioning scale [range 0 (high functioning/good health) until 100 (low functioning/ill health].

*Clinical characteristics of the anxiety disorder:* Clinical characteristics of remitted anxiety disorders were age of onset of the (first, in case of comorbidity) anxiety disorder, presence of ⩾2 lifetime anxiety disorders (yes/no), and number of years since remission of the last anxiety disorder.

*Psychiatric comorbidity:* To determine psychiatric comorbidity, the following disorders were assessed using the CIDI: current depressive disorder (i.e. major depression or dysthymia in the 12 months prior to baseline), remitted depressive disorder (a lifetime major depression or dysthymia but not a 12-month major depression or dysthymia at baseline), current alcohol use disorder (i.e. abuse or dependence in the 12 months prior to baseline) and remitted alcohol use disorder (a lifetime disorder but not a 12-month disorder at baseline).

*Health care for mental health problems:* Contact with general medical care or mental healthcare in the previous 12 months (at least one contact) was assessed. Treatment contact refers to at least one contact for emotional or addiction problems in the past 12 months. It was assessed with the question ‘In the past 12 months, did you visit any of the following professionals or institutions because of emotional or alcohol or drugs problems of your own?’ General medical care consisted of consultation with general practitioners, company doctors, social workers, home care or district nurses, physiotherapists or haptonomists, medical specialists or other professionals working within this care sector. Mental health care included psychiatrists, psychologists, psychotherapists or other psychiatric treatment. Use of psychotropic medications in the previous 12 months was also assessed.

### Statistical analysis

Characteristics of the study sample were analyzed using descriptive analyses (Table 1). With the Kaplan–Meier technique cumulative recurrence rates were estimated. These are somewhat higher than unadjusted recurrence rates, due to correction for censored data (Kleinbaum & Klein, 2005). For each year since remission, a recurrence rate was calculated by dividing the number of people who developed anxiety disorders, and thus relapsed, by the number still at risk. Cumulative recurrence rates were calculated by multiplying the recurrence rates reported up to that point. All respondents, including those not assessed at second follow-up, were included in these analyses. After the proportional hazards assumption was tested (i.e. shape of the failure function is the same for all levels of a particular predictor), and was not violated for any predictor, Cox regression analyses were performed to examine predictors for time to recurrence of any anxiety disorder. This method also corrects for censored data (Kleinbaum & Klein, 2005). Cox regression analyses adjusted for gender and age (hereafter indicated by univariate analyses) were conducted for all predictors separately. Subsequently, predictors with a *p* < 0.05 were entered into the multivariable model. Analyses were performed using STATA 12.1. Two-tailed testing procedures were used with 0.05 alpha levels in all analyses, except for the tests for the proportional hazards assumption, where alpha levels of 0.01 were used.

## Results

### Baseline characteristics

Baseline characteristics of 468 respondents with a remitted anxiety disorder are presented in Table 1. The mean age was 46.0 (s.e. = 0.54), and 64.5% of the respondents were female. The mean age of onset of the anxiety disorder was 19.9 (s.e. = 0.58). On average, respondents were 11.0 (s.e. = 0.0.47) years in remission at baseline. The majority of the respondents (85.0%) was diagnosed with only one anxiety disorder and almost half (47.9%) of the respondents had a current or remitted depressive disorder at baseline. Almost half (46.7%) of the respondents reported childhood abuse, had experienced a negative life event in the 12 months before baseline (51.5%) or has at least one biological parent with a history of psychopathology (45.3%). At baseline the following data were missing: age of onset (*n* = 3); not enough income to live on (*n* = 10); BMI (*n* = 30). Due to an abridged version of questionnaires used at *T*0 for a small part of the respondents, there were *n* = 8 missings on child abuse, parental psychopathology, neuroticism, chronic physical disorder, and service use for mental health problems.

### Recurrence during the 6-year follow-up period

**Fig. 1.**
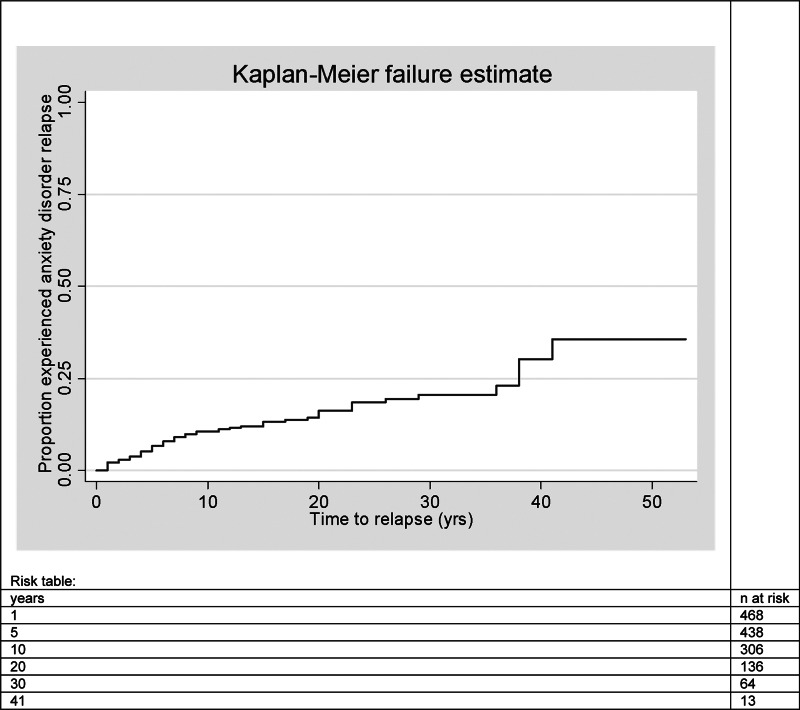
Kaplan–Meier curve of time to recurrence of anxiety disorders in respondents in remission from anxiety disorder at baseline (*n* = 468). The risk table presents the number of respondents at risk at the corresponding point in time.

Of the 468 respondents with a remitted anxiety disorder at baseline, 69 (14.7%) developed an anxiety disorder within the 6-year follow-up period. The estimated cumulative recurrence rate was 2.1% at 1 year, 6.6% at 5 years, 10.6% at 10 years and 16.2% at 20 years (Fig. 1). At 30 years the recurrence rate was 20.6% and after 41 years 35.7%, both with rather wide 95% confidence interval (CI) 15.7–26.7 and 23.1–52.3, respectively (Fig. 1).

### Predictors

In the univariate analyses which were adjusted for gender and age, the following variables predicted a shorter time to recurrence: younger age, lower and higher secondary education (reference category: higher professional, university), not having a paid job, not having enough income to live on, childhood abuse, parental psychopathology, higher neuroticism score, higher level of physical dysfunctioning, higher level of mental dysfunctioning, having experienced at least two anxiety disorders, and any remitted depressive disorder or any current depressive disorder at baseline (Table 2).

**Table 2.** Predictors of time to recurrence of anxiety disorder

	Univariate model	Multivariable model
Predictor	HR (95% CI)	HR (95% CI)
Sociodemographic characteristics		
Female gender	1.26 (0.76–2.09)	
Age at interview^a^	**0.76** (**0.59**–**0.97**)*	**0.66** (**0.50**–**0.87**)**
Education (Ref: higher professional, university)		
Lower secondary	**1.91** (**1.02**–**3.59**)*	1.31 (0.66–2.61)
Higher secondary	**1.88** (**1.02**–**3.48**)*	1.61 (0.83–3.11)
Living without partner	1.52 (0.93–2.50)	
No paid job	**2.01** (**1.20**–**3.38**)**	1.51 (0.87–2.62)
Not enough income to live on	**2.95** (**1.60**–**5.44**)***	1.50 (0.76–2.97)
Vulnerability characteristics		
Any childhood abuse	**1.99** (**1.21**–**3.30**)**	1.49 (0.89–2.50)
Any negative life event	1.40 (0.86–2.27)	
Parental psychopathology	**1.68** (**1.04**–**2.71**)*	**1.68** (**1.02**–**2.76**)*
Neuroticism^a^	**1.93** (**1.55**–**2.39**)***	**1.54** (**1.20**–**1.97**)***
Physical health and functioning		
Any chronic physical disorder	1.34 (0.82–2.19)	
BMI^a^	1.05 (0.83–1.33)	
Physical functioning scale^a^	**1.50** (**1.24**–**1.82**)***	1.17 (0.91–1.50)
Mental functioning scale^a^	**1.65** (**1.38**–**1.97**)***	1.24 (0.95–1.64)
Clinical characteristics of anxiety		
Age of onset anxiety disorder^a^	1.09 (0.82–1.46)	
Two or more anxiety disorders	**2.15** (**1.20**–**3.86**)*	1.78 (0.93–3.38)
Comorbid mental disorders		
Any mood disorder (Ref.: never)		
Remitted	**2.00** (**1.13**–**3.54**)*	1.64 (0.92–2.94)
Current	**6.47** (**3.39**–**12.33**)***	**2.93** (**1.49**–**5.76**)**
Any alcohol disorder (Ref.: never)		
Remitted	1.44 (0.74–2.82)	
Current	1.09 (0.33–3.68)	

HR, hazard ratio; Ref, reference category.

The reference category consists of those who did not develop an anxiety disorder during follow-up.

a Per standard deviation (s.d.) increase, s.d. age = 11.6; s.d. neuroticism = 2.7; s.d. BMI = 4.3; s.d. physical dysfunctioning = 19.2; s.d. mental dysfunctioning = 14.6; s.d. age of onset = 12.5.

Bold: Significant HR at the 0.05 level, two-sided test.

*p < 0.05; **: *p* < 0.01; ***: *p* < 0.001.

In the multivariable Cox regression analysis, in which all abovementioned significant variables as well as gender and age were entered simultaneously, four variables predicted shorter time to recurrence: younger age, parental psychopathology, higher neuroticism score and current depressive disorder (Table 2).

## Discussion

This study is one of the few general population-based studies investigating recurrence and predictors of recurrence of anxiety disorders within a national representative sample with a wide adult age range over a long follow-up period. The estimated cumulative recurrence rates were 2.1% at 1 year, increasing up to 10.6% at 10 years, 16.2% at 20 years, 20.6% at 30 years, and 35.7% after 41 years. Several variables predicted a shorter time to recurrence, of which younger age at interview, parental psychopathology, neuroticism, and a current depressive disorder remained significant in the age and gender-adjusted multivariable regression analysis.

As expected, the recurrence rates in this study were lower than rates found in studies among clinical samples (Bruce et al., 2005; Scholten et al., 2013, 2016), most likely because clinical samples predominantly represent more severe cases (Ten Have et al., 2013). When compared with studies in the general population (Calkins et al., 2009; Nay et al., 2013), recurrence rates from our study were comparable to the rates found in the 3-year follow-up study by Calkins et al. (2009). However, in this women-only-sample, both first onsets of anxiety disorders and recurrences were studied, and were added up in the results, and therefore the pure recurrence rate will be lower in their sample. Nay et al. (2013) found higher recurrence rates in people with panic disorders and agoraphobia (12.0% and 21.4% within 3 years, as compared to 2.1% at 1 year, 6.6% at 5 years in our study). It is not clear why these rates are much higher. It may have been of influence that Nay et al. (2013) also included recently remitted persons at baseline, while in our study only persons who were remitted at least 12 months before baseline were included. Therefore, our sample may have been more stable and healthy at baseline. It is also possible that panic disorders and agoraphobia are more often recurrent than other anxiety disorders. In this regard, previous studies are inconsistent, as this was also found in the HARP study (Bruce et al., 2005), but not in the NESDA study (Scholten et al., 2013). As far as we know, our study is the first to estimate the lifelong risk of recurrence following remission from an anxiety disorder in the general population. The results show that over a period of 41 years, 35.7% will experience a recurrence, sometimes even after many years of remission, suggesting that a substantial proportion of people remain vulnerable. These findings confirm the recurrent nature of anxiety disorders as derived from studies in clinical samples.

Many univariate predictors of recurrence were found, which can be of value in identifying those at the highest risk of recurrence. Most of these variables were also identified as predictors of recurrence in previous research in both clinical and general population samples (Bruce et al., 2005; Calkins et al., 2009; Fava, Zielezny, Savron, & Grandi, 1995; Nay et al., 2013; Pagano et al., 2007; Rodriguez et al., 2005; Scholten et al., 2013). However, the predictors ‘not enough income, no paid job’, having experienced child abuse, having experienced at least two anxiety disorders, and a remitted mood disorder were not assessed as predictors in previous general population based studies.

Multivariable analyses yielded a significant association for four predictors: younger age at interview, parental psychopathology, neuroticism, and a current depressive disorder. These variables are all factors indicating vulnerability. A younger age at interview predicting recurrence might reflect a younger age of onset, which was also found by Nay et al. (2013). The association between parental psychopathology and recurrence of anxiety corresponds with the generally recognized influence of heritability, nurture and environment on psychopathology in offspring (Hettema, Prescott, Myers, Neale, & Kendler, 2005). In addition, parental substance abuse has previously been described as predictor by Pagano et al. (2007), who found that a history of parental substance use disorder was a significant predictor of recurrence of social phobia and panic disorder. Neuroticism predicting recurrence accords with earlier observations which showed that neuroticism makes people vulnerable to developing a first episode of anxiety disorders and of recurrence (Calkins et al., 2009; De Graaf, Bijl, Ravelli, Smit, & Vollebergh, 2002). The finding that neuroticism was an independent predictor in our study may partly be caused by the fact that neuroticism and anxiety disorders share genetic and environmental determinants (the common cause model), which appear to play an important role in this prospective association (Ormel et al., 2013). Related variables, such as anxiety sensitivity, which is considered a specific aspect of the more general construct of neuroticism (Sexton, Norton, Walker, & Norton, 2003), proved to be predictive of recurrence in other studies (Scholten et al., 2013; Taylor et al., 2016). It is plausible that someone who has recovered from an anxiety disorder, who continuously responds fearful to stress and to anxiety symptoms, is more likely to redevelop an anxiety disorder.

The results of this study further show that a current depressive disorder (at baseline) is a predictor of recurrence of anxiety, which is in line with results from previous studies (Bruce et al., 2005; Fava et al., 2001b; Nay et al., 2013). Nay et al. (2013) also reported that a previous history of major depressive disorder (MDD) is a predictor of recurrence of PD/PDA, and Fava et al. (1995) found that the absence of a depressed mood increased the probability for patients with PD to remain in remission.

Compared to other studies (Rodriguez et al., 2005; Scholten et al., 2013; Taylor et al., 2016), this study also found poor functioning to be predictive of recurrence, but this did not remain a significant predictor in our multivariable analysis however, and this may be due to a different composition of the set of predictors in other studies.

### Strengths and limitations

A strength of this study is that, to our knowledge, it is the first longitudinal population-based study estimating time to recurrence of anxiety disorders over extended periods of time in a large representative general population cohort. Another strength is that a broad set of putative predictors was used to analyze predictors of recurrence.

This study also has some limitations worth mentioning. First, the sample size of this study was not large enough to study the predictors of the different anxiety disorders separately, and anxiety disorders were therefore studied as one group. It would have been informative to know whether separate anxiety disorders have different relapse rates and different predictors of recurrence. However, transitions between anxiety disorders occur frequently in recurrent anxiety disorders, and this may reflect a more general underlying vulnerability for anxiety in general (Scholten et al., 2013). Therefore, looking at all anxiety disorders as a category is more informative.

Second, since our study used retrospective diagnoses it is important to take the risk of underreporting of lifetime mental disorders into account (Moffitt et al., 2009). Consequently, in the selection of our sample, people may have been mistakenly excluded, causing selection bias. Assuming that underreporting is most likely in less severe cases with lower chances of recurrence, our findings may show too high recurrence rates. In the same vein, telescoping bias may have played a role in our study in reporting the number of years since remission of the last anxiety disorder. In telescoping, respondents may recall more events as having occurred in the most recent period (forward telescoping) and fewer in the more distant past (backward telescoping). This phenomenon tends to cause an overestimate of the number of events in the recent period. In this study this may have caused shorter time to recurrence. These biases are unfortunately inherent to this type of research.

Third, anxiety sensitivity, which was found to be an important predictor in previous studies (Calkins et al., 2009; Scholten et al., 2013; Taylor et al., 2016), was not included in NEMESIS-2, and could therefore not be studied, although we did study the related variable neuroticism.

### Clinical implications

This study shows that recurrence of anxiety in the general population occurs regularly and extends over a lengthy period of time. This means that, even after years of remission, people are still at risk of recurrence. This requires lifelong alertness to recurrence of anxiety disorders, especially for those at the highest risk. The healthcare system should be structured in such a way that people who are at the highest risk of having a recurrence of an anxiety disorder can be identified, monitored and have quick access to mental health care in case of recurrence. Identifying these people can be challenging since they come from the general population, and will most likely not be monitored in mental health care when they have recovered. General practitioners (GPs) most often constitute the first point of medical contact and act as gatekeepers to the rest of the health care system, including mental health care. Therefore, the GP could be the person playing an important role in the monitoring and early recognition of anxiety disorder relapses. Unfortunately, the GP may not have enough time or knowledge to accommodate this relapse prevention task. Clinicians and decision makers could strive for the appointment of other first-line health care personnel, specially aiming at prevention and simple treatment tasks in primary mental health care. Depending on the healthcare system, such personnel (e.g. nurses) could work in close collaboration with the GP or work in the GP practice. Indeed, in The Netherlands primary care nurse practitioners have been appointed to the GP practice for certain somatic disorders (for example diabetes mellitus), but also for common mental disorders. But we have to bear in mind that not all people have regular consultations on mental health problems with their GP. In our sample only 12% of the respondents had had contact with a GP about mental problems in the past 12 months. However, people visit their GP for other reasons, and during these visits GPs could take the initiative to bring up the subject of recurrence of anxiety themselves. In case of recurrence, easy access to consultation with the GP or with mental healthcare should be available. Furthermore, patients who are treated in mental health care in the acute phase of the anxiety disorder should be informed about the risk of recurrence and make a relapse prevention plan, to increase patients' awareness of signs of relapse, and to agree on the steps to be taken in case of recurrence. Patient associations and public health campaigns could also play a role in educating recovered people. Furthermore, our findings provide leads for interventions to prevent recurrence. We identified younger age, parental psychopathology, higher neuroticism score and current depressive disorder as independent predictors of recurrence, of which neuroticism and depressive disorders are treatable. Treatment of depression and neuroticism in remitted anxiety disorder patients can therefore contribute to preventing recurrence.

The combination of making recovered patients and health care providers aware of the risk of relapse and its predictors, as well as providing monitoring and easy access to consultation, could contribute to the prospect of a lasting recovery for people in the general population who are at risk of recurrence of anxiety disorders.
